# Enteroviruses: epidemic potential, challenges and opportunities with vaccines

**DOI:** 10.1186/s12929-024-01058-x

**Published:** 2024-07-15

**Authors:** Minne Jartti, Malin Flodström-Tullberg, Minna M. Hankaniemi

**Affiliations:** 1 Virology and Vaccine Immunology, Faculty of Medicine and Health Technology, Tampere University, Tampere, Finland; 2 Department of Medicine Huddinge and Karolinska University Hospital, Karolinska Institutet, Stockholm, Sweden

**Keywords:** Enterovirus (EV), EV outbreaks, Vaccine development, EV surveillance

## Abstract

Enteroviruses (EVs) are the most prevalent viruses in humans. EVs can cause a range of acute symptoms, from mild common colds to severe systemic infections such as meningitis, myocarditis, and flaccid paralysis. They can also lead to chronic diseases such as cardiomyopathy. Although more than 280 human EV serotypes exist, only four serotypes have licenced vaccines. No antiviral drugs are available to treat EV infections, and global surveillance of EVs has not been effectively coordinated. Therefore, poliovirus still circulates, and there have been alarming epidemics of non-polio enteroviruses. Thus, there is a pressing need for coordinated preparedness efforts against EVs.

This review provides a perspective on recent enterovirus outbreaks and global poliovirus eradication efforts with continuous vaccine development initiatives. It also provides insights into the challenges and opportunities in EV vaccine development. Given that traditional whole-virus vaccine technologies are not suitable for many clinically relevant EVs and considering the ongoing risk of enterovirus outbreaks and the potential for new emerging pathogenic strains, the need for new effective and adaptable enterovirus vaccines is emphasized.

This review also explores the difficulties in translating promising vaccine candidates for clinical use and summarizes information from published literature and clinical trial databases focusing on existing enterovirus vaccines, ongoing clinical trials, the obstacles faced in vaccine development as well as the emergence of new vaccine technologies. Overall, this review contributes to the understanding of enterovirus vaccines, their role in public health, and their significance as a tool for future preparedness.

## Enteroviruses

Enteroviruses (EVs) constitute a genus of viruses belonging to the *Picornaviridae* family that consists of more than 280 viruses capable of infecting humans. The genus includes 15 species – the 12 *Enterovirus* species (A-L) and three *Rhinovirus* species (RV A-C) [1, 2]. The three poliovirus serotypes (poliovirus 1-3) are found within the species C *Enterovirus* and the rest of the enteroviruses are commonly referred to as non-polio enteroviruses (NPEVs). Seven out of the 15 NPEV species (EV A-D and RV A-C) infect humans, and NPEVs are among the most common human pathogens found worldwide, with infections particularly frequent in children. Examples of commonly circulating NPEVs are rhinoviruses, coxsackievirus (CV) A and B, EV-D68 and EV-A71 [1, 2].

**Fig. 1:**
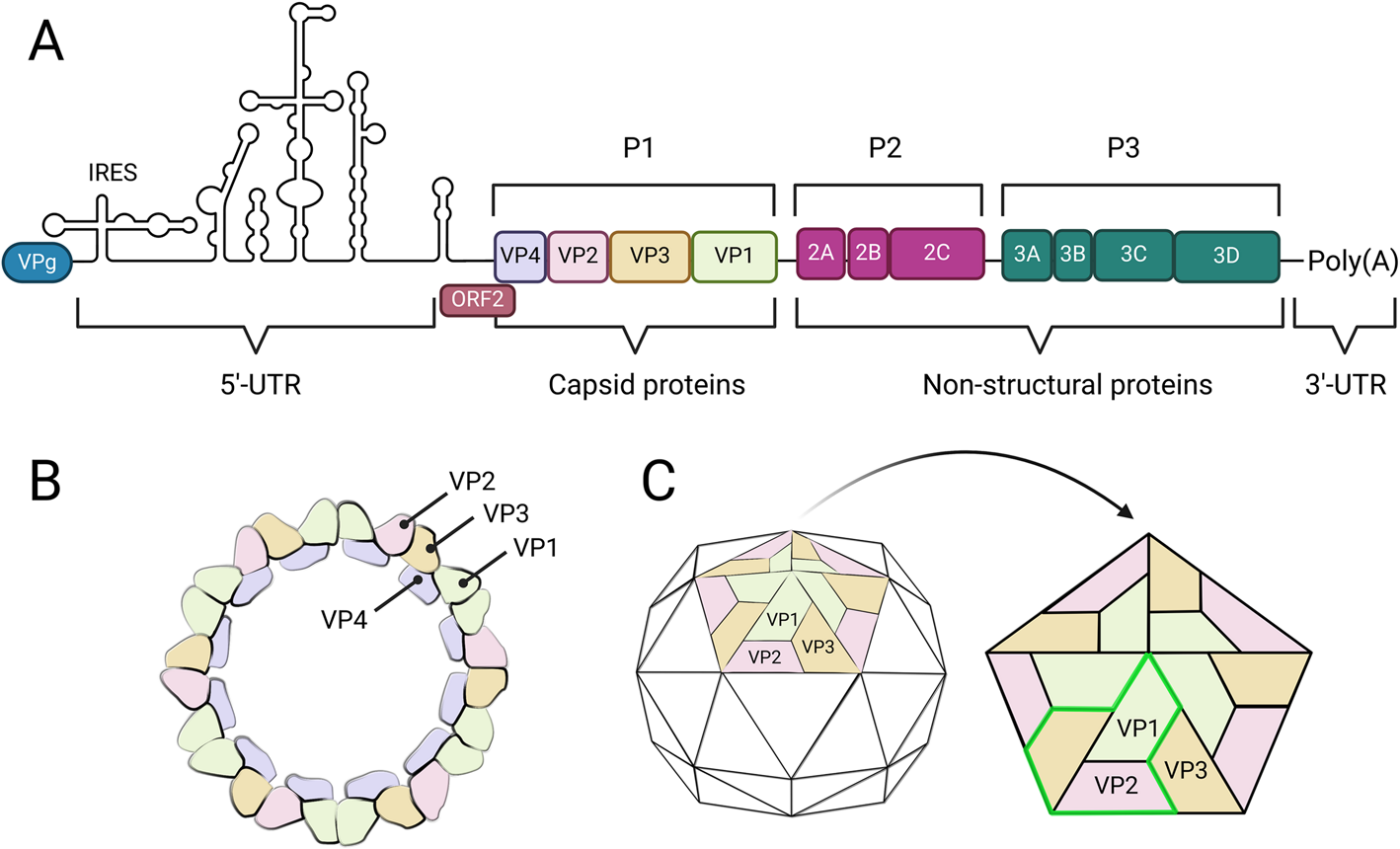
Enterovirus genome organisation and capsid structure (not to scale). **A** enterovirus genome encodes a single polyprotein (regions P1-P3) comprising a main open reading frame (ORF). Additional recently discovered [3, 4] ORF2 overlaps with the main ORF at the 5’ end. The ORF2 is not found from rhinoviruses or EV-D68 [4] **B** enterovirus capsid is formed by the four viral structural proteins VP1-VP4 of which the VP4 is located at the inner capsid, **C** while VP1-VP3 form a pseudo T=3 symmetry unit (highlighted with neon green) which assembles into pentamers 12 of which in turn form the outer capsid

EVs are non-enveloped viruses with a single stranded (ss) RNA molecule of about 7500 bases surrounded by an icosahedral capsid consisting of 60 copies each of the four viral proteins VP1-4 (Fig. 1). EV virions are very stable and most EVs have a strong resistance to acidic environments (down to pH 3.0). This enables many EVs to infect and replicate in the gastrointestinal tract, and faecal-oral spread is the primary route of transmission, but many EVs can also transmit via oral and respiratory droplets originating from infected individuals.

**Fig. 2:**
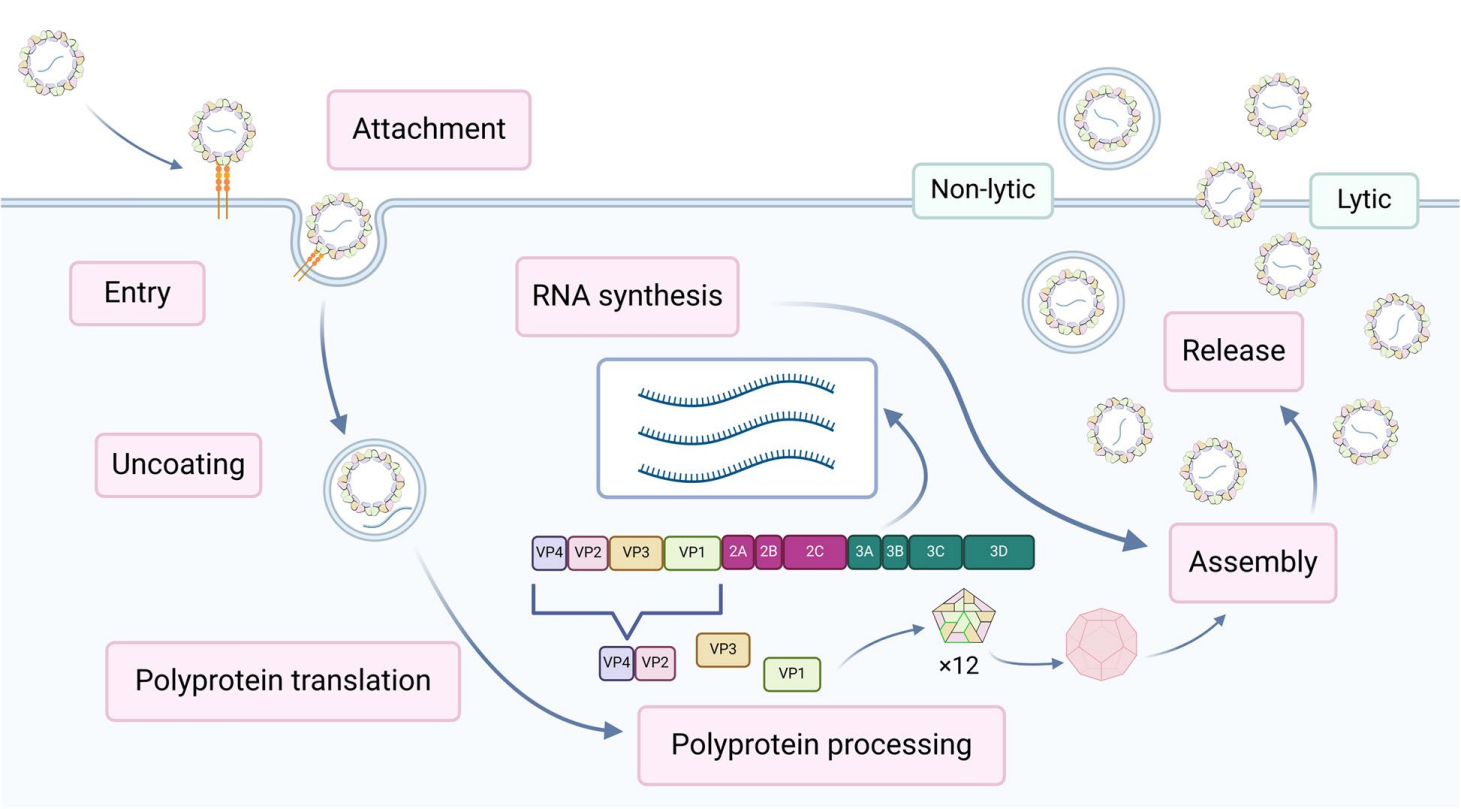
Enterovirus replication cycle (schematic representation). After receptor attachment and internalization by endocytosis, the genome is uncoated. As positive stranded RNA viruses, enteroviruses utilise host-cell machinery in genome replication – the replication takes place in a replication organelle, which are composed of cellular membranes prompted by the infection. After assembly the mature virions are released by either lytic or non-lytic pathways

The EV replication cycle begins with virion binding to a cellular receptor followed by endocytic uptake of the virion. The virion then delivers the viral RNA genome to the cytosol where it is translated into a polyprotein. The polyprotein is proteolytically processed by viral proteases 3C and 2A into structural (capsid) and non-structural proteins, including the viral polymerase. Non-structural proteins then induce the formation of membrane structures (replication organelles) where genome replication takes place (Fig. 2). Subsequently, capsid proteins and genomic RNA self-assemble into virions that exit the host cell via cell lysis or within extracellular vesicles [5]. Transmission via filopodia has also been described [6, 7].

While most NPEV infections result in mild respiratory illness, such as the common cold, some can lead to more severe illnesses. These include acute flaccid myelitis (AFM, a paralyzing illness), myocarditis, hand-foot-and-mouth disease (HFMD) and neonatal sepsis [8]. NPEV infections are also responsible for more than 50% of aseptic meningitis cases [9]. It is important to note that some complications of enterovirus infections may not be evident until years after the initial infection. For example, coxsackie B virus (CVB) infections are known to cause myocarditis, which can progress to chronic dilated cardiomyopathy [10] months or even years after acute infection [11]. NPEVs that affect the central nervous system and heart pose a particularly heavy burden on the health care system [8, 11]. Additionally, CVB infections have been associated with the autoimmune disease type 1 diabetes [6]. Thus, the disease and health care impacts of enterovirus infections are substantial (Table 1).

**Table 1:** Enterovirus (EV) disease-associations (excluding rhinoviruses). Abbreviations: Coxsackievirus B (CVB), Coxsackievirus A (CVA), Echovirus (E)

**Disease**	**Virus**	**References**
Acute flaccid paralysis (AFP)	EV-A71, EV-D68, CVA1, CVA20, E11, E33 EV-B73, EV-B93, EV-94	[12–16]
Non-polio acute flaccid myelitis (AFM)	EV-D68, EV-A71, EV-B93, EV-D94, EV-D111	[12, 13, 17, 18, 18–20]
Paralytic poliomyelitis	Polio1, -2 and 3	[21]
Meningitis	CVB1-6, EV-A71, E2, E9, E18, E30, E3	[22–25]
Encephalitis	CVB1-6, EV-A71, E11	[25, 26]
Myocarditis	CVB1-6, EV-6, E9, E11, E30	[27–29]
Pancreatitis	CVB3, CVB4, EV-A71	[30–32]
Neonatal systemic illness	CVB1-6	[30]
Hand, Foot and Mouth Disease (HFMD)	CVA1, CVA6, CVA9, CVA10, CVA16, CVA22, CVA24, CVB1, CVB2, CVB3, CVB4, CVB5, EV-A69, EV-A71, EV-D68, E3, E4, E5, E6, E9, E11, E15, E16, EV99	[33–37]
Sepsis	CVB1, CVB3, CVA16	[38, 39]
Type 1 diabetes (T1D)	CVB1, CVB4	[40–42]
Bronchiolitis, Pneumonia	CVA6, EVD68, EV-A71	[43–46]
Herpangina	CVA6, CVA9, CVA10, CVA16, CVA20, CVA22, CVA24, CVB2-5, E1, E6, E7, E9, E21 EV-A71,	[34, 37]
Influenza like illness	CVA6, CVA10, CVB1, CVB2, EV-A71, EV-D68	[34, 47]
Severe paediatric respiratory illness	EV-D68	[47]

Since NPEVs are single-stranded RNA viruses with high mutation and recombination rates, this increases the risk of new pathogenic strains emerging in the future. Therefore, we think that it is critical to develop effective vaccines and adaptable vaccine platforms that can be rapidly deployed to respond to emerging or re-emerging enteroviruses. To our opinion it is particularly important to prioritize vaccine development concerning EVs associated with the most severe disease manifestations such as acute flaccid paralysis (AFP), AFM, meningitis, myocarditis, neonatal systemic illness as well as neurological complications. Thus, vaccines against emerging viruses (such as EV-B93, EV-D94, EV-D111) as well as re-emerging viruses (such as EV-A71, EV-D68, CVA1, CVA20, E11, E33, CVB1-6, EV-6, E9 and E30) should be actively progressed.

Additionally, the development of safe and effective antiviral drugs should be pursued – antivirals are an active area of research, especially against EV-A71, and are reviewed elsewhere [48–57]. Although five EV inhibitors targeting the EV capsid surface have been evaluated for safety and efficacy in clinical trials, a majority of the inhibitors were found to cause unwanted side effects and failed to meet their clinical endpoints [58]. Notably, inhibitors may lead to the development of drug-resistant strains; however, it has been observed that resistant strains typically exhibit lower levels of fitness than wild-type strains [58]. This review focuses on current and new enterovirus vaccines and vaccine technologies in development for poliovirus and other NPEV vaccines, while rhinovirus vaccines have been covered in reviews elsewhere [59, 60].

## *Enterovirus* outbreaks are common

In recent years, several outbreaks of both polioviruses and NPEVs have occurred. While Afghanistan and Pakistan are the only countries where wild-type poliovirus remains endemic, in July 2024, there were 35 so-called ‘outbreak countries’ across five continents, implying the ongoing risk of imported wild-type poliovirus or the emergence of circulating vaccine-derived poliovirus (cVDPV) [61]. Poliovirus outbreaks resulting from mutations of the attenuated oral polio vaccine (OPV) are reported periodically, as in 2022, when cVDPV was detected in wastewater samples from New York, London and Jerusalem. Two cases of polio-related paralysis caused by cVDPV were reported in Israel and in the United States [62, 63]. According to the World Health Organization (WHO), one in 200 polio infections can lead to irreversible paralysis (typically affecting the legs) [64]. Therefore, every paralysis case suggests that many other people have been infected with polio and that the virus is circulating more widely. This is particularly alarming given that endemic poliovirus has largely been eradicated, and suboptimal vaccination coverage in at-risk populations poses a threat to the global polio eradication initiative.

NPEV outbreaks also pose a risk to human health. Between 2015 and 2017, 66 different NPEV types were identified circulating in 24 EU/EAA countries, leading to 68 deaths, 77 cases of paralysis and over 3000 neurological infections, 30% of which were paralytic [8]. EV-D68 gained attention in the United States in 2014 when it caused over 1300 confirmed cases, primarily among children, resulting in five deaths. The outbreak was also linked to several cases of polio-like syndrome [65]. Since then, EV-D68 has emerged as a biennial epidemic in the United States and Europe [66, 67]

After the 2014 outbreak of EV-D68 in North America, a study revealed that a majority of the children admitted to the hospital with AFM still presented persistent motor deficits after one year [68] and that EV-D68 continued to circulate after the initial outbreak [69]. A significant increase in the number of EV-D68-positive patients was again observed in September 2022 by the Johns Hopkins Hospital system, with 28% of patients requiring hospital admission and 49% of those requiring intensive care [70]. A similar unexpected outbreak of EV-D68 occurred in Finland during the same period and led to the hospitalization of several children [71]. This increase in EV-D68 cases is believed to account for the increase in cases of AFM every two years since 2014 [72–74]. The occurrence of AFM, characterized by sudden paralysis, follows the seasonal circulation pattern of enteroviruses, particularly that of EV-D68. Furthermore, a study from the United Kingdom revealed that the incidence of EV-D68 infections in young children was notably higher in 2016 compared to 2006, suggesting changes in population immunity, virus antigenicity, transmissibility, or cellular tropism [75]. The change in the clinical presentation of EV-D68 has been hypothesized to be caused by mutations, potentially allowing broader receptor binding, thus leading to increased virulence, transmissibility and infections at a younger age [76–79].

In addition to EV-D68, unexpected peaks of severe and fatal entero- and parechovirus infections were documented in the United States during 2022 [80]. Similarly, an increase in CVB-induced severe cases of myocarditis in neonates was reported in the United Kingdom between June 2022 and April 2023 [81]. Several European countries also reported numerous cases of echovirus 11 (E11)-related sepsis and meningoencephalitis in newborns between 2022 and the summer of 2023, some of which were fatal [82, 83]. Aseptic meningitis is one of the severe sequelae, caused by several different EVs (Table 1 above), such as CVBs and EV-A71. Enterovirus associated meningitis outbreaks have recently been reported in 2023 in Iraq (caused by both bacterial and enteroviral infections) [84], in 2019 in Mayotte French Comoros Island [85], and 2018-2019 in South Africa [86] A study found that in the United States between 2011-2014 almost 60% of studied meningitis and encephalitis cases were found to have enterovirus as an etiological agent [87]. In 2018, an increase in echovirus 30 (E30) infections associated with meningitis/meningoencephalitis were observed in Denmark, Germany, the Netherlands, Norway and Sweden [88], and a 2022 CVB2 outbreak caused meningoencephalitis in Israeli children [89], while in 2016 an outbreak of aseptic meningitis caused by E30 broke in Inner Mongolia – an autonomous region of China [90]. Cases of HFMD and neurological illness causing enteroviruses in Japan between 2012-2019 are reviewed at [91], while the circulation of non-polio enteroviruses in EU and EEA countries – resulting in e.g. HFMD, myocarditis and death – are studied at [8].

EV-A71 frequently causes outbreaks in East- and Southeast Asia [34, 92–96] (reviewed recently at [97]), and these outbreaks have been associated with several deaths in children [97, 98]. In 2011-2012 an EV-A71 outbreak resulted in fatalities in Vietnam’s largest outbreak of HFMD [99]. EV-A71 is not limited to Asia, as it has also been detected in European countries. In 2001, EV-A71 was found in Scottish blood donors [100]. Sporadic cases and epidemics of EV-A71 infections have been reported across Europe [101–107]. In 2016, Catalonia was hit by an outbreak that later spread across Spain, resulting in 57 children suffering from severe neurological disease instead of HMFD [17, 108]. The largest outbreak of EV-A71 in the Americas was observed in 2018, during which 43 children showed symptoms of meningitis, encephalitis, AFM or seizures [18].

EV-A71 is a major cause of HFMD along with CVA16, but the disease is caused by several EVs as indicated in Table 1 above. CVA6 has been proposed as a new emerging pathogen causing HFMD globally, as reviewed in [109]. HFMD is particularly troublesome in Asia-Pacific region, causing widespread outbreaks typically affecting young children [110, 111]. While the disease caused in majority of cases is mild, due to its prevalence HFMD contributes to mortality which interestingly was also found to be the major driver in bringing up the disability-adjusted life years (DALYs) associated with HFMD [112]. Thus it is suggested that HFMD associated DALYs could significantly be brought down by vaccination lowering mortality [112].

The closely associated enteroviruses EV-B93, EV-D94, and EV-D111 were suggested to be the cause of AFM outbreaks in both Egypt and the Democratic Republic of the Congo in the early 2000s [12]. Similarly, EV-B93 was identified in a patient who presented with AFP in Tibet in 1999 [13]. According to phylogenetic analysis, that particular EV-B93 strain had undergone recombination with EV-B107 [13]. Although it was discovered through seroepidemiology that EV-B93 had not caused an epidemic in Tibet, the EV-B93 strains identified in Tibet exhibited temperature resistance and prognosticative virulence, suggesting the potential for a large-scale outbreak [13].

A recent analysis revealed that EV-D111 strains isolated from human and non-human primate samples were not phylogenetically distinct, suggesting recent zoonotic transmission [113]. Evidence of intertypic genetic recombination events between EV-D111 and EV-D94 was also discovered, indicating a shared replication site in infected hosts [113]. Both EV-D94 and EV-D111 induce cytopathic effects in L20B cells commonly used to detect polioviruses [113]. It was hypothesized that this could lead to false-positive poliovirus detection, particularly in Central Africa, where EV-D111 circulates and is a key region for poliovirus eradication [113]. Taken together, these findings emphasize the continuous emergence of new enterovirus species and their zoonotic relatedness and potentially pathogenic nature, which call for epidemic preparedness. Although none of these NPEV outbreaks have yet led to disease on a global scale and have not been identified via clinical or laboratory-based enterovirus surveillance outside Africa, polio has taught us that enteroviruses possess both emerging and re-emerging potential.

## The lack and need for *enterovirus* epidemic preparedness

Epidemic preparedness requires a robust surveillance system to detect outbreaks early, sufficient healthcare infrastructure and clear plans on how to act in outbreak situations. Key elements of effective preparedness efforts include political commitment, public health communication plans, sustained funding of public health infrastructures as well as research and development of vaccines and therapeutic treatments. Additionally, international collaboration between countries, organizations, and health agencies facilitates a coordinated global response to prevent and manage outbreaks.

Rapid detection, characterization and control of circulating enteroviruses requires a proactive system based on regular sampling of appropriate patient cohorts and general surveillance on a global scale [21], which is currently lacking. Apart from EV-A71 vaccines used in China and Thailand, there are no approved vaccines for NPEVs. NPEV antivirals are also lacking [58, 114, 115]. As a result, the preparedness for NPEV outbreaks and epidemics is inadequate.

A recent article identified several knowledge gaps in enterovirus research that are crucial to pandemic preparedness [116]. These include, for example active surveillance strategies and comprehensive models of transmission, which are necessary for accurate disease outbreak predictions and evaluating interventions. Because enteroviruses are so common, many of them co-circulate which allows for recombination and thus leads to increased transmission potential [117–123]. Although the zoonosis of EVs has been poorly characterized, host transmission barrier is considered high for EVs. However, many EVs, including poliovirus, echoviruses, CVA and CVB have been shown to also infect non-human primates [124–130] and a novel viral recombinant between poliovirus and coxsackievirus displaying AFP symptoms was isolated from chimpanzees in sub-Saharan Africa [131]. Therefore, we think that integrating EV pandemic preparedness with One Health Concept would be crucial in enhancing overall public health outcomes against EVs.

EV-D68 has been suggested as a test case to establish an immunological surveillance program and develop countermeasures for future outbreaks, as the expected EV-D68 spike during 2020 was avoided, likely due to non-pharmaceutical interventions for COVID-19, but left behind a susceptible population [132]. In the United States, the National Institute of Health Vaccine Research Center’s PREMISE (Pandemic REsponse REpository through Microbial and Immune Surveillance and Epidemiology) program aims to address the problem of surveillance by 1) conducting immune analysis to detect reactivity against potentially pandemic viruses, 2) identifying immunogens for vaccine development and 3) developing monoclonal antibodies for prevention and therapy [133]. Similarly, in Europe, the European Non-Polio Enterovirus Network (ENPEN) aims to develop standardized protocols for hospital-based surveillance, diagnosis, detection and reporting of enterovirus-associated infections to establish the true burden of NPEV infections in Europe [134], while in Asia-Pacific region the Asia-Pacific Network for Enterovirus Surveillance (APNES) has been established with an analogous function [135].

Epidemic preparedness efforts against enteroviruses vary greatly between countries, largely reflecting the epidemiological situation and healthcare resources. The Global Polio Eradication Initiative (GPEI) is a collaborative effort aimed at eradicating polio worldwide through immunization, surveillance, and public health interventions [136]. Led by national governments and six core partners (the WHO, Rotary International, CDC, UNICEF, Bill & Melinda Gates Foundation, and Gavi, the Vaccine Alliance), the GPEI is a public‒private partnership. The WHO declared the European region free of wild-type poliovirus in 2002. However, the recent transmission of OPV-derived poliovirus in the United States, Europe and the Middle East [137], as well as the movement of refugees from war zones of Pakistan and Afghanistan, has increased the risk of new poliovirus outbreaks (either vaccine-derived or wild-type) globally. For example, a recent study conducted in Ethiopia revealed that only one-third of Ethiopian children receive all polio vaccine doses required for efficient protection against poliomyelitis [138]. Factors contributing to low vaccination coverage included limited access to health care facilities, dissatisfaction with vaccination services, low parental education and fear of vaccine-related side effects. As such, the study highlights the need for improved vaccination strategies among at-risk populations as well as the need to address barriers in both access and inequity in coverage. In addition, this underscores the need for continued vaccine vigilance as well as work to reduce vaccination hesitancy and barriers that lead to a lack of vaccination as a key approach to epidemic preparedness.

As part of the commitment to GPEI’s polio eradication strategy [139], the CDC and WHO have recommended enterovirus surveillance to 1) detect and control outbreaks, 2) conduct complete virological investigations and research for at least 80% of all AFP cases (surveillance indicators found at [140]), and 3) gather data for long-term public health planning. Several countries, including the United States [141], Australia [142], Germany [143], and 25 other European countries [144], have established surveillance systems that collect information on cases associated with enterovirus infections.

Current enterovirus surveillance systems primarily rely on passive methods, which involve detecting enteroviruses in diagnostic patient samples by PCR or serology. This approach is essential because of the large number of distinct enterovirus serotypes that can infect humans and the fact that the diseases they cause are often mild. However, in certain cases, additional laboratory surveillance of poliovirus and NPEV has been conducted among high-risk populations. This supplementary surveillance is necessary to meet the requirements set by the WHO, which include tracking the emergence of vaccine-derived polioviruses, the reappearance of wild polioviruses, or the disappearance of all vaccine-related strains [145].

To effectively eradicate polio and halt the transmission of NPEVs, the development of broadly reactive enterovirus vaccines that can be mass produced, supplied, and administered without the assistance of healthcare professionals would be the most effective approach. Vaccines should also be available to people in low- and middle-income countries and could thus realistically achieve global coverage. Investments in vaccine development are therefore crucial for epidemic preparedness efforts.

## Existing *enterovirus* vaccines

Vaccination plays a crucial role in controlling and eradicating infectious diseases. The global battle against polio has witnessed remarkable advancements due to poliovirus vaccines. These vaccines have led to a 99.9% reduction in polio cases over the past three decades. There are two different types of poliovirus vaccines, the inactivated whole-virus vaccine (IPV) and the OPV. OPV has been instrumental in the fight against polio. It consists of a live-weakened form of poliovirus, is administered orally, replicates in the gut, and induces better mucosal immunity compared to IPV, which is administered intramuscularly. Moreover, OPV is more cost-effective and easier to administer, making it the preferred option in low-income countries.

OPV has enabled significant progress in the global eradication of polio, as in the past it was administered in now polio-free countries. However, the final stages of eradicating the disease have proven to be challenging, mainly due to persistent outbreaks of circulating OPV-derived polioviruses (cVDPVs). While wild-type polioviruses, particularly type 1 polioviruses, still pose a threat in countries including Afghanistan and Pakistan, outbreaks of all three poliovirus types can still occur. Currently, type 2 cVDPVs are the most prevalent variant of vaccine-derived viruses [146]. To overcome this, the WHO declared the Polio Eradication and Endgame Strategic Plan (2013-2018) in 2013. The plan outlined various goals, one of which was to cease the use of the oral polio vaccine containing all three poliovirus serotypes. This process began with the removal of the type 2 poliovirus from the OPV. In April 2016, the trivalent vaccine (tOPV), which contained all three serotypes, was replaced with the bivalent vaccine (bOPV), containing only serotypes 1 and 3. Currently, many countries use a combination of bOPV and IPV (types 1, 2 and 3) as part of their routine vaccination programs [147]. However, due to the eradication of wild-type poliovirus in high-income countries, OPV is no longer used for safety reasons.

Based on a database query on adisinsight.spinger.com (accessed 23.8.2023), there are currently 23 combination vaccines containing IPV on the market. These vaccines also include other antigens, such as diphtheria, tetanus, pertussis, and hepatitis B [148–150]. They typically contain inactivated virus, and their formulation varies based on location and intended age group. Combination vaccines have helped reduce the need for multiple injections and have contributed to widespread polio vaccine coverage. Well-known stakeholders such as Sanofi, AJ Vaccines, Intravacc, Sinovac, WHO, KM Biologics, and Mitsubishi have registered or preregistered three multivalent polio vaccines for use against multiple diseases.

As EV-A71-induced disease has become a major public health problem in China [151], three inactivated and alum-adjuvanted EV-A71 vaccines were introduced in China between 2015 and 2016. In 2022, the first EV-A71 vaccine was licenced in Thailand. EV-A71 vaccines are inactivated whole-virus vaccines that offer cross-protection among EV-A71 subgenotypes [151, 152], but these vaccines have not yet received regulatory approval outside of Asia. To be used worldwide, there is a need for global harmonization in terms of vaccine production, quality control, and standardization. Notably, the currently available EV-A71 vaccines are designed to target the C4 sub-genotype, which is the most prevalent sub-genotype circulating in China. If the Chinese vaccine quality regulations would comply globally, the safety and efficacy of these vaccines would need to be tested in the new intended target populations since the dominant EV-A71 strains are different outside of China [152]. The efficacy of the three existing vaccines after two vaccinations is very high, at more than 90%, and remains so at the two-year follow-up [151]. Candidate vaccines containing the B4 and B5 genogroups are in development elsewhere but have not yet reached the licensing stage [151].

## *Enterovirus* vaccines in clinical trials

**Fig. 3:**
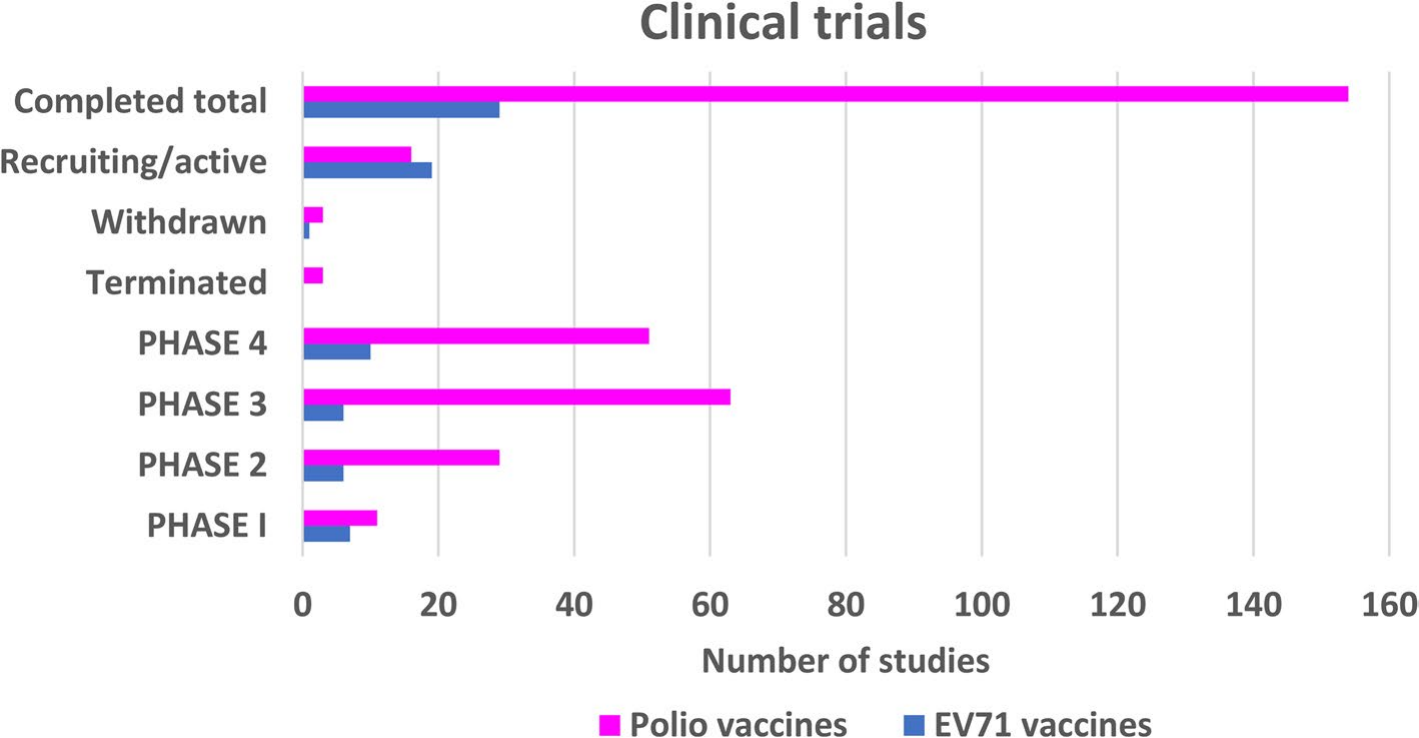
The enterovirus vaccine landscape in clinical trials. Majority of enterovirus vaccine clinical trials are conducted for polio- and EV-A71 vaccines. In addition to those, single phase I clinical trial has been completed for CVB1 vaccine [79] (not shown in the figure). Based on www.clinicaltrials.cov search

**Fig. 4:**
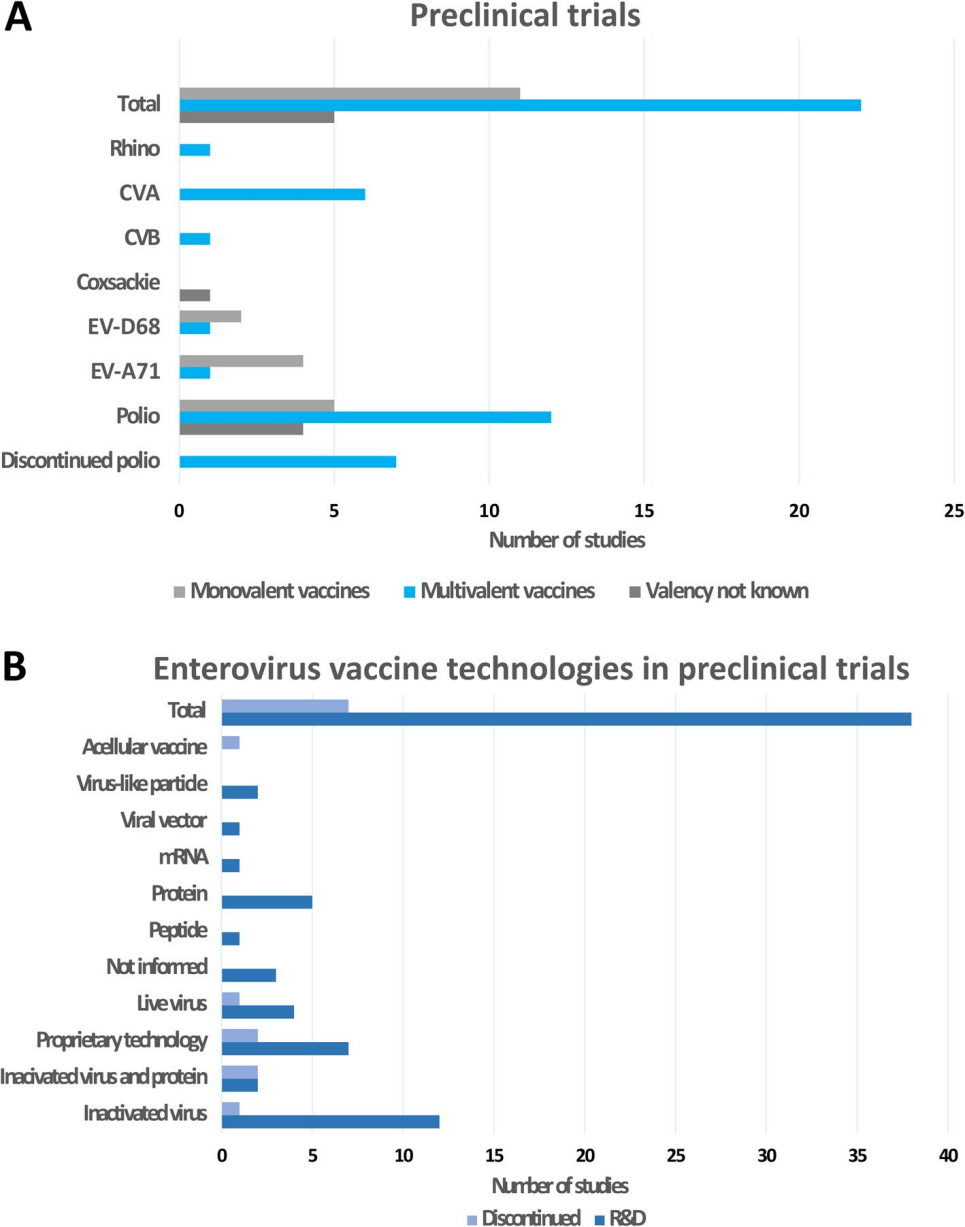
The enterovirus vaccine landscape prior to clinical trials. The figure depicts enterovirus vaccines in **A** preclinical trials and **B** enterovirus vaccine technologies in preclinical trials. Based on www.adisinsight.com search

To map the current enterovirus vaccine landscape, we conducted a systematic search of all enterovirus vaccine trials through ClinicalTrials.gov (accessed 9.1.2024). This investigation showed that polio and EV-A71 vaccine trials still dominate the field (Fig. 3). There are 154 polio and 29 EV-A71 vaccine clinical trials, that have been completed, of which 51 and 10 are completed phase 4 studies for polio and EV-A71 vaccines respectively (Fig. 4). Of the 154 polio vaccine trials, 8 involve trials studying the efficacy of IPV and OPV, when administrated sequentially, whereas 120 involve trials on IPV and 23 studies on OPV (in mono- or multivalent formulations). Three of the vaccine trials involve comparison of a fractional IPV dose administered intradermally to a full dose administered intramuscularly. These studies involve the IPV vaccine shortage associated to the withdrawal of type 2 OPV in April, 2016, when trivalent OPV (containing types 1, 2, and 3) was replaced with bivalent OPV (containing types 1 and 3 [153]). Of the 29 completed EV-A71 vaccine trials 21 studies involve usage of vero-cell produced inactivated virus vaccine and three studies involve usage of human Diploid Cell (KMB-17) produced inactivated virus vaccine, whereas one study compared the efficacy of the vaccines produced in Vero vs. KMB-17 cells. Five of the studies did not reveal the vaccine technology or the production host. The completed polio vaccine clinical trials reflect the situation in the marketed polio vaccines. Most completed clinical trials for enterovirus vaccines have been done for combination vaccines where polio has been formulated together with diphtheria, tetanus and pertussis (e.g. study sponsored by GSK [154]). These clinical studies are necessary for evaluating whether antigenic interference occurs and for determining optimal vaccine dose for different groups of people. In addition to the polio and EV-A71 vaccine clinical trials, a first-in-human phase 1 study was recently conducted with a multivalent CVB inactivated virus vaccine. This vaccine, based on our vaccine platform and promising preclinical studies [155–160], targets several strains of CVBs associated with islet autoimmunity and type 1 diabetes (T1D). The phase 1 study suggested that the vaccine is well tolerated and elicits virus-neutralizing antibodies in vaccinated individuals [161].

To enhance the safety of the oral poliovirus vaccine, a research team genetically modified type 2 poliovirus, increasing the stability of the new type 2 OPV (nOPV2) vaccine [162]. The team hypothesized that the modified weakened virus is less likely to mutate, evolve, or cause infection. After the assessment of phase II and phase III clinical trial data, as well as additional data on safety, efficacy, and manufacturing quality, the nOPV2 vaccine was granted emergency use listing (EUL) approval by the WHO in 2020. This marked a significant milestone, as it was the first ever vaccine to receive such approval [163]. Since the approval of the EUL, more than 600 million doses of the nOPV2 vaccine have been administered in 28 countries, primarily in response to polio outbreaks. Therefore, nOPV2 is currently in the wider roll-out phase, and with the increased use of nOPV2 in the last 2 years, both the incidence of cVDPV2 and the intensity of cVDPV2 transmission have decreased [164]. The nOPV2 was designed to prevent reversion to the virulent form but has failed in that respect. Seven cVDPV2 cases of nOPV2 originating from 61 paralytic cases and 39 environmental surveillance (sewage) samples were detected in six African countries during August 2021–July 2023, although the incidence of reversion is still approximately 10 times lower than that of the original type 2 monovalent oral polio vaccine (mOPV2) [165]. This demonstrates the high mutation and recombination capacity of enteroviruses and thus their epidemic potential.

The same research group modified the remaining two strains of poliovirus types 1 and 3 in OPV. Genetic modifications similar to those for type 2 poliovirus were made to these strains, resulting in the creation of new vaccine candidates. These vaccine candidates have shown great promise in mouse studies, particularly in terms of their immunogenicity when administered as monovalent and multivalent formulations [166]. Genetically modified novel live weakened type 1 and type 3 oral polio vaccines (nOPV1 and nOPV3) are currently undergoing phase I and II clinical studies to ensure that they are both effective and do not revert to dangerous forms in humans. Like for nOPV2, the first trials are being conducted to evaluate the safety, immunogenicity, shedding and genetic stability of the vaccine candidates in IPV-primed adults before the vaccines can be tested in a larger adult and adolescent (> 15 y of age) population or in young children or infants (in this order). If approved, the nOPV1 and nOPV3 vaccines can be combined into bivalent or trivalent combinations with nOPV2 and contribute to polio eradication.

In the case of EV-A71 vaccines, five are combination vaccines, where inactivated EV-A71 virus is formulated together with one or several of the following vaccines: inactivated CVA16 virus, split influenza virus vaccine, hepatitis B vaccine, meningococcal polysaccharide vaccine, mumps and rubella, Japanese encephalitis vaccine, or weakened measles virus. For completed clinical studies on EV-A71 combination vaccines, the seroconversion rates of antibodies against EV-A71 have been more than 94%, and combination vaccines have induced non-inferior responses to monovalent EV-A71 vaccines [167].

Most of the clinical trials on EV-A71 vaccines have focused on the three inactivated vaccines that are currently marketed in China. These vaccines differ from each other in terms of the vaccine strain, manufacturing cells (human diploid cells vs Vero cells), and antigen dose. However, clinical studies have demonstrated that the immunogenicity of these vaccines is nearly the same in children between the ages of 6 and 35 months [152]. This suggests that EV-A71 vaccines are equally effective in stimulating an immune response in young children, which is a critical target population for EV-A71 vaccination.

As EVs have caused several outbreaks over time globally and the number and diversity of EVs is high, each human has contracted several symptomatic or asymptomatic EV-infections during their lifetime. Therefore, if a new EV vaccine is developed and analyzed in clinical trials, assessing its efficacy becomes possible by examining correlates of protection. These correlates include vaccine-specific antibody levels in samples from the vaccinated individuals. However, because of the existing immunity against EVs circulating globally and locally, the efficacy of the vaccine needs to be determined e.g. by comparing the antibody-levels from pre- and post-immunization samples to see if the new vaccine has been able to induce immunity. If the new vaccine is approved for clinical use, we recommend prioritizing both young and elderly individuals for vaccination because infections are most common and most severe at the extremes of age, where the immune system is the most vulnerable.

## *Enterovirus* vaccines in research and development phase

To date, virtually all existing vaccine technologies and approaches for the development of enterovirus vaccines have been explored (Table 2). The vaccine technologies differ in terms of production method and the immune response inducing antigen. According to a database query made from adisinsight.spinger.com (accessed 23.8.2023), the majority of enterovirus vaccines in the research and development phase are EV-A71 and poliovirus candidate vaccines. However, there have also been developments in CVA, CVB and EV-D68 virus vaccines of varying valencies (Fig. 4A). In total, there were 22 multivalent vaccines, 11 monovalent vaccines, and five vaccines with unknown valency in the research and development phase. Combination vaccines have been considered a solution to the issue of increased numbers of injections during single clinic visits. Previous evaluations have been conducted on the safety and efficacy of vaccines containing diphtheria, tetanus, pertussis, hepatitis B, *Haemophilus influenzae* and type B with polio. While the formulation of a combination vaccine encompassing seven vaccines has been discontinued, there are several similar multivalent vaccine formulations in clinical use. In terms of enterovirus vaccine technologies (Fig. 4A), 12 vaccines were based on inactivated viruses, and seven utilized proprietary technologies. However, a total of eight different technologies have been employed for enterovirus vaccine development (Fig. 4B). Because the number of EVs infecting human is high, but the cross-reactivity of the immune response against EVs is low, multivalent vaccine formulations containing vaccine antigens against the most prevalent and severe disease causing EVs should be developed. We and others have shown previously that the multivalent vaccine approach is feasible as immunity against the six CVBs [158] or 50 different rhinoviruses [168] could be successfully achieved with an approach combining 6 or 50 inactivated viruses in one vaccine formulation. However, the vaccines were based on traditional inactivated viruses, which is not a feasible approach against several EVs. Therefore, new vaccine technologies combining an optimal formulation of vaccine antigens should be developed in order to produce a multivalent EV vaccine. Efforts to develop vaccines against CVB [169], enterovirus A species and EV-A71 [170, 171] have been extensively reviewed elsewhere [169–173]. As noted, essentially all currently existing vaccine technologies have been employed to develop enterovirus vaccines, as demonstrated with the examples of vaccine development studies below.

**Table 2:** Vaccine technologies that have been used in enterovirus vaccine development

***Vaccine technology***	***Antigen***	***Pros***	***Cons***
***Inactive virus***	Whole virus	Efficient activation of immunity [158]	Expensive to produce. Risk of insufficient inactivation of the virus
***Weakened virus***	Mutated virus or recombined with another weakened virus	Efficient mucosal, humoral and T-cell responses [174]	Risk of reversion back to virulent form and causing new epidemics [175]. Not recommended for risk groups
***Virus-like particle (VLP)***	VP1-4 co-expressed with 3CD	Cost-effective, safe, retains virus structure [172, 176]	Immunogenicity may need to be enhanced with adjuvants [177]
***Recombinant protein / subunit / peptide***	VP1-4/subunits/ peptides from VP1-4	Safe, easy to produce [178]	Low immunogenicity, cost-efficiency varies
***Vector***	VP1-4	Efficient immunogen [179]	Pre-existing immunity for the vector [180]
***DNA***	VP1	Quick and easy to produce [181]	Efficient administration method is needed, risk of genome integration
***mRNA***	VP1-4 and 3CD	Quick and easy to produce, high neutralizing antibody titres [182]	Short immune response, low mucosal immunity
***Exosome***	VP1	Efficient T-cell and cytotoxic T-cell response [183]	Limited availability of studies about these vaccines, low production rate

### Inactivated virus vaccines

Inactivated virus vaccines are a ‘traditional’ vaccine technology and have been in use for over a century. The technology is based on production of inactive viruses where the infectivity of the virus is destroyed either by a chemical (such as formalin [155] or beta-propiolactone [184]) or irradiation. We have developed inactivated virus vaccines for the six CVBs and demonstrated their safety and efficacy as monovalent [155–160] and multivalent [158] vaccines in animal models. Based on these studies, a double-blind randomised placebo-controlled Phase I trial for multivalent CVB vaccine targeting serotypes associated with type 1 diabetes has been completed [161]. Inactivated virus vaccines have been developed also for CVB3 (reviewed in [169]), CVA5 [185], CVA6, CVA10 [184, 186], CVA16 [187], CVA2 [188] and EV-D68 [189]. In 2001 researchers studied different approaches for developing a EV-A71 vaccine based on passive immunization [178]. In lethal challenge studies the antibodies transferred from mothers immunized with inactivated virus provided most protection for the suckling mice when the level of protection was compared to subunit based and DNA vector based vaccines [178]. Although it seems unlikely that inactivated virus vaccines could solve EV problem, the advantage of the technology is existing manufacturing infrastructure in the countries that have active vaccine industry.

### Weakened virus vaccines

Weakened virus (also known as attenuated) vaccines are the other ‘traditional’ vaccine technology and based on reducing the infectivity of the pathogen by either mutations or deletions. Often weakened virus vaccines are developed by serial passaging in cell cultures over multiple generations until the virulence of the virus has been weakened, while still being immunogenic. Weakened virus vaccine against EV-A71 [190, 191] and CVB3 (reviewed in [169]) have been produced, while attenuating mutations that render CVA6 less virulent have been recently described [192]. Although weakened virus vaccines have proven to be very efficient against poliovirus as well as many other pathogens, more safe and applicable vaccine technologies should be developed.

### Virus-like particle vaccines

Virus-like particles (VLPs) are a vaccine technology based on producing vaccines by using one or several structural proteins (capsid proteins) of the virus. The proteins are produced recombinantly, typically in insect-, yeast-, or bacterial cells, but also mammalian cells are used. Experimental VLP based vaccines have been developed for EV-A71 [193], CVA6 [194], CVA10 [195], CVA16 [176] and EV-D68 [196, 197] as well as for CVB1 and CVB3 which we have developed [172, 173]. Additionally, immunogenic but unstable poliovirus-like particles have been produced previously [198, 199]. To combat the instability and improve the immunogenicity of these VLPs, chemical crosslinking and genetically engineered disulfides have been utilized, and these stabilized particles have been shown to generate high levels of neutralizing antibodies in animal models [200–205]. The most distinct advantages of VLP-based vaccines are their safety and applicability against different pathogens, as well as the cost-effective manufacturing processes that can be optimized for several different expression hosts.

### Recombinant protein- subunit and peptide vaccines

Like in the case of VLP-based vaccines, in the recombinant, subunit or peptide-based vaccine approaches virus components are produced recombinantly, for example in insect-, mammalian-, or bacterial cells. These types of vaccines are considered to be much safer for at-risk populations compared to traditional whole virus technologies. In the case of EV-A71 vaccines based on VP1 recombinant protein, DNA-vector and inactivated virus, the VP1 recombinant protein vaccine provided protection against lethal challenge only at the lowest challenge dose. However, the subunit vaccine demonstrated better efficacy than the DNA vector [178]. Although the inactivated EV-A71 vaccine elicited a stronger immune response compared to the other vaccines tested, protein-based vaccine technologies show promise for future vaccine development [178]. Recombinant subunit vaccines developed against CVB3 are reviewed in [169].

### Vector vaccines

Virus vector vaccines utilise a second virus – often adenovirus – as a delivery vector or platform for the target antigen. For virus vector vaccines the virus genome is mutated, and it cannot replicate. Virus vector technologies have been explored for the development of EV-A71 vaccine utilizing adenovirus as a vector [179]. The researchers inserted the EV-A71 *P1* and *3CD* genes into the E1/E3-deleted adenoviral genome and in immunogenicity studies the vaccine candidates were shown to provide immunity against EV-A71 in a mouse challenge model [179]. Vector vaccines developed against CVB3 are reviewed in [169].

### DNA vaccines

In DNA-vector based vaccine technology the DNA vector is produced by inserting the viral target gene (e.g. VP1) into eucaryotic expression vector (e.g. pVAX1). Upon vaccination the DNA vector enters host cell nuclei for mRNA transcription, and the antigen is produced in target cells. In the EV-A71 vaccine study, a VP1 DNA vaccine was tested along with inactivated virus and subunit vaccine, and the DNA vector based vaccine provided some protection in lethal challenge studies, but overall performed weakest compared to inactivated virus and VP1 recombinant protein vaccines [178]. Another study also looked at constructing a EV-A71 VP1 DNA vector vaccine using pVAX1 expression vector into which the VP1 gene was cloned [181]. The VP1 DNA vector vaccine elicited an immune response in a mouse model, but further development improving the expression and immunogenicity of the vaccine would be required [181]. DNA vaccines developed against CVB3 are reviewed in [169].

### mRNA vaccines

For mRNA-based approaches, mRNA of the target antigen gene(s) is synthesized and packed into lipid nanoparticles. Upon vaccination the mRNA utilises host cell machinery in antigen production, inducing an immune response. mRNA vaccines developed against CVB3 are reviewed in [169].

### Exosome vaccines

Exosomes are extracellular vesicles of endosomal origin, and they are produced by almost all cell types [206]. In exosome-based vaccine development strategies exosomes are produced by target virus producing cell lines, and the exosomes are released into the supernatant. Recently, an exosome based antigen delivery strategy was tried for CVB3 VP1 vaccine, and was shown to enhance resistance to CVB3 induced myocarditis in a mouse model – more interestingly the study found the exosome based vaccine to be more potent compared to recombinant protein VP1 subunit vaccine the group had created previously [183, 207].

## What has been learned from immune responses elicited during *enterovirus* infection and preclinical and clinical *enterovirus* vaccine trials

Enterovirus infections induce the production of virus-neutralizing antibodies, which provide long-term protection against reinfection by the same serotype. Sera have therefore long been used to define virus serotypes, which is also the reason why infection with one serotype does not confer protection from another serotype. Therefore, it is currently believed that effective enterovirus vaccines should elicit serotype-specific antibodies. However, partial neutralization of related enterovirus serotypes has been observed when studying serotype-specific sera [208], and several monoclonal antibodies (mAbs) that bind and neutralize multiple poliovirus serotypes have been isolated from infected humans [209]. These antibodies exhibit varying affinities for the three poliovirus serotypes, and two of them, which have cross-serotype capabilities, can block infection by poliovirus serotypes 1 and 2 in cell culture, while the third poliovirus serotype remains unaffected by these antibodies. These data indicate that poliovirus antibody cross-neutralization involves binding to highly conserved structures within the canyon that bind to the cellular receptor [209] and demonstrate that perhaps not all virus-neutralizing antibodies are solely serotype specific. Although polio vaccine coverage has increased over 90% in the US, biennial outbreaks causally associated to AFM have been caused by EV-D68 for unknown reason [210]. Likewise, epidemiological and surveillance studies demonstrated that CVA6 has replaced EV-A71 as the major cause of hand, foot and mouth disease in Nanchang China following the deployment of an EV-A71 vaccine, suggesting that vaccination against one enterovirus may lead to replacement by another [211].

Research has demonstrated that neutralizing antibody levels above a protective threshold are essential, and effective memory CD4+ T-cell responses confer long-term protection against enteroviruses [212]. Although the humoral immune response is important for controlling enterovirus infections, it has also been suggested that inadequate cellular immunity is associated with more severe clinical outcomes of these infections. Decreased cellular immunity and lower levels of interferon-gamma (IFN-γ) have been found to correlate with the severity of EV-A71 infection [213]. Whether cellular immunity (e.g., CD8+ T-cell responses) and/or IFN-γ contributes to robust immunity against enterovirus infections after vaccination is not fully understood. However, studies on IPV and OPV have provided some interesting insights and accumulating evidence suggest the importance of CD4+ and CD8+ T-cells for optimal protective immunity against EVs induced by vaccination [174].

The immune responses elicited by IPV and OPV are different. IPV provides systemic humoral immunity and only modest intestinal immunity to all three types of poliovirus [214]. Vaccination does not prevent infection but stops poliovirus from reaching the central nervous system, thus preventing paralytic poliomyelitis. The presence of virus-neutralizing antibodies in plasma/serum after IPV vaccination correlates with protection against paralytic disease. In contrast to IPV, OPV induces both systemic and mucosal humoral immunity, providing additional local protection at the gastrointestinal tract, which is effective in interrupting infection and transmission of the virus. Furthermore, OPV vaccination seems to induce long-term CD4+ and CD8+ cytotoxic T-cell responses [174]. Cross-reactive T cells have been shown to contribute to host protection against viral infections, including those induced by influenza and SARS-CoV-2 [215], and it is possible that a similar phenomenon occurs after OPV vaccination, which could provide partial protection against other enteroviruses. Consistent with this, several studies have demonstrated that vaccine induced CD8+ T-cell priming may improve the efficacy of immunization against influenza [216] and SARS-CoV2 [217, 218]. Research has indicated that there are cross-reactive B- and T-cell responses to conserved enteroviral epitopes in healthy individuals vaccinated against polio [219, 220]. Moreover, an immune response to a conserved enteroviral B-cell epitope on the major capsid VP1 protein has been associated with a decreased risk of cardiovascular disease [220]. Additionally, human T cells have been found to recognize group-specific enteroviral antigen(s) [221–223]. Identifying motifs that elicit cross-reactive B and T cells and incorporating these residues into future vaccines could provide a measure of protection against EVs with epidemic potential or accelerated clearance. How the cellular immune response contributes to robust intestinal immunity and adequate virus-specific IgA levels in the mucosa and in the serum remains to be fully understood. A recent study investigating how CVB affects human β cells and anti-CVB T-cell responses demonstrated that CVB-infection induces limited antiviral CD8+ T-cell responses [223]. Therefore, we anticipate that inducing CD8+ T-cell responses through vaccination might be beneficial in promoting antigen cross-presentation, but also for being essential in promoting the early protective effects during the first two weeks after the initial vaccination, when neutralizing antibody levels are still too low for protecting against the virus.

The proximity of enterovirus T-cell epitopes to B-cell antigenic sites in human poliovirus suggests that there may also be a requirement for spatial proximity of T- and B-cell epitopes to attain efficient protection against enteroviruses [222]. Nevertheless, there are notable gaps in the knowledge surrounding what constitutes an optimal immune response to enteroviruses and how this response can be induced by vaccines. According to studies on polio- and EV-A71-vaccinated or infected individuals, it seems that the optimal immune response requires balanced humoral, cellular and mucosal immune responses in addition to correct spatial T- and B-cell epitope proximity, which might also be needed.

## Challenges and opportunities in *enterovirus* vaccine development

Traditional vaccine production methods, particularly for enteroviruses, pose several challenges. One issue is safety concerns, as vaccines based on whole-virus particles may not be adequately inactivated, and live-weakened virus vaccines may revert to a virulent form. Another challenge for inactivated whole-virus particle vaccines is the time-consuming and costly production process, which is unlikely to meet the demand during a global pandemic. The successful growth of large quantities of virus particles is required, but this is difficult or even impossible for certain EVs due to their inefficient growth in standard cell culture systems. Our own unpublished research suggests that the production of vaccines against some enteroviruses, such as CVA6, CVA10, CVA16, EV-A71, and EV-D68, face challenges in this regard. Additionally, traditional vaccine production methods can lead to the loss of immunogenicity, and the risk of the introduction of mutations is high when viruses are cultured in cells (our unpublished results). In the case of an inactivated virus vaccine, there is also uncertainty about its ability to induce sufficient intestinal immunity to protect populations where faecal-oral transmission is predominant. Another major limitation of traditional enterovirus vaccines is their specificity for a particular serotype, although some cross-protection has been found. Currently, the inactivated EV-A71 vaccines produced in China demonstrate high effectiveness, with vaccine efficacy exceeding 90% against the C4 sub-genogroup included in the vaccine. In addition to T lymphocytes, neutralizing antibody responses have been shown to be equally important in the protection of mice from EV-A71 infection [212]. Similar to poliovirus vaccines, EV-A71 clinical trials have shown that although high levels of neutralizing antibodies could be sufficient for protection, poor cellular immunity might increase the risk of vaccine failure, severe neurological complications and death [212]. Therefore, the results from EV-A71 mouse infection models have indicated that humoral immunity protects mice from lethal EV-A71 challenge, but for optimal immunity against enteroviruses, better cellular immunity is desirable [212]. Studies have demonstrated that the cross-neutralizing activity of EV-A71 vaccines (based on subgenotype C4) can provide broad cross-protection against different EV-A71 subgenotypes, but ongoing research and development are essential to address the challenges posed by the genetic evolution and immunogenicity of EV-A71 vaccines [224].

Evidence for the ability of cross-serotype anti-enterovirus antibodies to block infection have been gathered since 1920s when monkeys were infected with three different poliovirus isolates and were challenged with infection by heterologous isolates [225–227] and some cross-protection was found. Moreover, monoclonal antibody A12 has been shown to bind all three serotypes of poliovirus [228]. However, the binding to different serotypes is not similar and this antibody blocks the infection of cells in culture for serotypes 1 and 2 but not for serotype 3. Moreover, a recent study demonstrated that anti-enterovirus antibodies can inhibit infections by heterologous enteroviruses, when sera from mice immunized with the EV-D68 protected cells from infection poliovirus type 1/Mahoney. The same study demonstrated that sera from mice immunized with poliovirus type 1/Mahoney protected cells against infection by EV-D68, EV-A71 and HRV1A and weakly against EV-D94 [219].

Given the large number of enterovirus serotypes capable of infecting humans and the high likelihood of new strains emerging, it is practically impossible to develop vaccines against all enterovirus serotypes using traditional methods. Therefore, to better prepare for future epidemics, it is crucial to establish vaccine platforms that can promptly address the demand for adaptable and rapid responses during outbreaks. These vaccines should be easy and quick to produce, have a high degree of safety, and have a well-characterized composition that can be easily modified to meet specific requirements in the case of re-emergence of enteroviruses or the appearance of new pathogenic strains. Furthermore, these vaccines must be cost-effective to manufacture and supply. For this purpose, the development of vaccine technologies that incorporate scalable production hosts and scalable manufacturing and purification methods is necessary. These advancements will enable vaccines to be quickly mass-produced and readily available. Additionally, the design of these vaccines should prioritize safety, effectiveness, and the ability to be transported without the need for ultralow temperatures. Like marketed vaccines, multivalent vaccines in the development phase are administered parenterally, which means that these vaccines are not very effective at inducing mucosal immune responses. As a result, while these vaccines can help alleviate the severity of enterovirus disease, they typically cannot completely stop the spread of the virus. Considering this limitation, it is important to shift the focus from the exploitation of traditional vaccine technologies and immunization routes to new technologies and ways to administer vaccines (e.g., mucosal administration). OPV has shown that vaccine administration to mucosal surfaces can create immunity at the site of virus entry, thereby effectively stopping the infection. If such a vaccine was to be translated into clinical use, achieving a vaccination rate of approximately 60-70% of the human population could potentially create herd immunity.

A change in the enterovirus vaccine development landscape also requires a change in the interface of the academic and industrial vaccine development sectors since bringing promising candidate vaccines from preclinical studies into clinical trials is very expensive and requires expertise in vaccine manufacturing process optimization, quality control and regulatory aspects. Researchers at universities should consider these aspects while the development work is ongoing. The patenting of new technologies or manufacturing processes will also facilitate the transition from preclinical to clinical studies, as buying or licencing intellectual property rights is key for the pharmaceutical industry to make investments.

## Conclusions

Vaccination has long been recognized as the most effective method for preventing the spread of viral diseases on a global scale. It has played a significant role in reducing morbidity and mortality associated with various infectious diseases. However, the lack or ineffectiveness of vaccines has been identified as a major contributing factor to the emergence and propagation of epidemics and pandemics. Poliovirus still maintains its status as a Public Health Emergency of International Concern (PHEIC) – declared by the WHO in 2014 – despite the significant progress made in the eradication program over the past 35 years. While outbreaks of circulating variant poliovirus have decreased in the Eastern Mediterranean Region, both Afghanistan and Pakistan remain endemic countries for wild poliovirus.

There are more than 280 known enterovirus serotypes, several of which cause acute infections in humans, occasionally with short- and long-term life-threatening complications. Enteroviruses can mutate, recombine and cause devastating epidemics. However, the only available vaccines are against the three poliovirus serotypes and EV-A71. Furthermore, current enterovirus vaccines are mainly serotype specific, associated with high production costs and, in the case of poliovirus, are at risk of the emergence of circulating vaccine-derived viruses. The absence of adaptable vaccine technologies is a significant barrier to combatting re-emerging and new enterovirus strains. In addition, many low- and middle-income countries face substantial challenges in establishing and maintaining vaccine manufacturing capacity. This limitation further exacerbates the global problem of vaccine shortages and leaves vulnerable populations without access to life-saving immunizations.

The development and production of vaccines require a high level of expertise, resources, and infrastructure. With respect to enteroviruses, it is critical to develop new vaccine platforms that can rapidly address the demand for new vaccines during outbreaks. These vaccines must be easy and quick to produce, have a high degree of safety, and have a well-characterized composition that can be easily modified to meet specific requirements in the case of re-emergence of enteroviruses or the appearance of new pathogenic strains. In addition, these vaccines must be cost-effective to manufacture and deliver. With such measures, we will be in a better position to react to future enterovirus epidemics.

## Data Availability

References for this review were identified through a search of PubMed for articles published by use of the terms “enterovirus” and “vaccine” or any specific enterovirus serotype, e.g. “Enterovirus 71”. Other relevant references were identified from key online sources (e.g., WHO, CDC, clinicaltrials.gov, adisinsight.com). Only articles published in English were included.
